# Meta-analysis of cyathostomin species-specific prevalence and relative abundance in domestic horses from 1975–2020: emphasis on geographical region and specimen collection method

**DOI:** 10.1186/s13071-020-04396-5

**Published:** 2020-10-12

**Authors:** Jennifer L. Bellaw, Martin K. Nielsen

**Affiliations:** grid.266539.d 0000 0004 1936 8438 M. H. Gluck Equine Research Center, Department of Veterinary Science, University of Kentucky, Lexington, KY USA

**Keywords:** Cyathostomin, Prevalence, Relative abundance, Critical test, Diagnostic deworming, Necropsy

## Abstract

**Background:**

Cyathostomins infect virtually all horses, and concomitant infections with 10 or more species per horse is standard. Species-specific knowledge is limited, despite potential species bias in development of disease and anthelmintic resistance. This is the first meta-analysis to examine effects of geographical region and cyathostomin collection method on reported composition of cyathostomin communities.

**Methods:**

Thirty-seven articles published in English in 1975 or later, in which adults of individual species were systematically enumerated, were included. Seven regions; North America, South America, eastern Europe, western Europe, northern Europe, southern Africa, and Oceania, and three cyathostomin collection methods; (i) standard necropsy recovery from the large intestine, (ii) critical test collection from post-treatment feces and necropsy, and (iii) diagnostic deworming recovery solely from post-treatment feces, were considered. Generalized mixed linear models analyzed the effects of region and collection method on species-specific prevalence and relative abundance. Species richness was analyzed by mixed linear models.

**Results:**

Definitively, the most prevalent and relatively abundant species were *Cylicocyclus nassatus* (prevalence = 93%, relative abundance = 20%), *Cylicostephanus* (*Cys.*) *longibursatus* (93%, 20%), and *Cyathostomum catinatum* (90%, 16%). A bias toward horses with high infection intensities and cyathostomin collection from feces resulted in North American critical tests and eastern European diagnostic deworming overestimating the species-specific prevalence and underestimating the relative abundance of rare/uncommon species compared to respective intra-regional standard necropsies. North American critical tests underestimated species richness due partially to identification key errors. Inter-regional standard necropsy comparisons yielded some species-specific regional differences, including a significantly higher *Cys. longibursatus* prevalence and relative abundance in North America (92%, 33%) than in eastern Europe (51%, 7%) (*P* > 0.0001). Localization of critical tests to North America and diagnostic deworming to Eastern Europe precluded expansive ‘region by collection method’ interaction analyses.

**Conclusion:**

We provide substantial data to inform study design, e.g. effect and study size, for cyathostomin research and highlight necessity for method standardization and raw data accessibility for optimal *post-factum* comparisons.
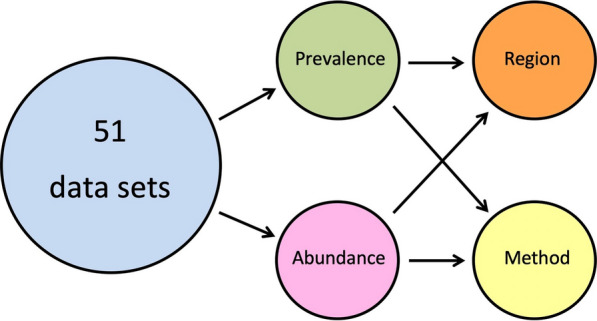

## Background

Strongylid parasites of horses (Nematoda: Strongylidae) comprise a vast complex of 50 currently recognized species [1]. Cyathostomins, referring collectively to 40 Cyathostominae and seven non-migratory Strongylinae species, infect virtually all grazing horses with prevalence frequently approaching 100% [2–5]. Concomitant infection with 10 or more cyathostomin species per individual horse is the norm, rendering infections inherently complex [6–8].

Historically, the Strongylinae, most particularly *Strongylus vulgaris*, were regarded the most pathogenic gastrointestinal nematodes infecting equines, but species of this subfamily were observed to decline dramatically during the 1980s, and cyathostomins have since then been recognized as prominent parasitic pathogens of horses [9, 10]. Cyathostomins cause the rare but often fatal clinical syndrome, larval cyathostominosis [11, 12], and exhibit emerging or widespread resistance to all currently available anthelmintic drug classes [13]. Cyathostomins are thusly the most important gastrointestinal parasites of horses weaning age and older [14, 15]. Despite this, species-specific research on basic cyathostomin biology and ecology or on population and epidemiological dynamics involved in clinical disease and anthelmintic resistance is wanting. The major cause of this is limitations of available diagnostic tools [16, 17].

Only adult stage specimens can be morphologically identified to species, albeit with significant training and expertise [1]. In equine cyathostomin research, adult specimens are primarily collected *via post-mortem* methods. Standard necropsies are the most common and are often opportunistic, utilizing euthanized horses from veterinary hospital cases and abattoirs [2, 18, 19]. Therein, cyathostomin adults are recovered from digesta within the large intestine. Standard necropsies generally accommodate larger sample sizes than methods specific to anthelmintic efficacy trials and reduce ethical concerns of maintaining horses for this sole purpose. However, standard necropsies are not adequate for anthelmintic trials in which enumeration of specimens both expelled within feces and remaining in the horse are important [20]. In the critical test method, horses are first anthelmintically treated, and expelled adults are collected from feces during a post-treatment interval. After which, horses are necropsied and adults collected from digesta as above [21]. Critical tests are labor intensive and require maintenance of horses for this specific purpose, resulting in few and small-scale studies. A third *post-mortem* method, the controlled test, entails systematic necropsy and parasite collection from matched treatment and control groups after a post-treatment interval. This necessitates prolonged maintenance of study horses and larger group size than with critical tests [20]. To address ethical constraints of terminal studies, an *ex-vivo* method has been used to monitor anthelmintic resistance outside of efficacy trials [22]. In this diagnostic deworming method, expelled adults are recovered from feces following adulticidal anthelmintic treatment without subsequent necropsy [22]. While this method theoretically allows larger-scale studies, processing massive amounts of feces still significantly limits study size and application. This method is not ideal as dependence on collection post-adulticidal anthelmintic treatment theoretically biases toward collection of susceptible species [20]. In the absence of cross-validation, the potential impact of parasite collection method on study outputs, i.e. species richness (the number of species encountered), species diversity (the number of species and relative abundance of each), and species-specific prevalence, is a major caveat to inter-study comparisons that are common practice especially in monitoring anthelmintic resistance.

The goal of this meta-analysis was to begin parsing baseline cyathostomin community dynamics from potential study design biases. We collated published species-specific prevalence and relative abundance at the adult meta-population level to analyze the influence of geographical region and adult specimen collection method on community composition that may critically affect study outcomes and confound interstudy comparisons. Specifically, we aimed to (i) substantiate anecdotal regional differences in species-specific prevalence and relative abundance and overall species richness, (ii) investigate the impact of adult specimen collection method, e.g. standard necropsy, critical test, and diagnostic deworming, on these outcomes in lieu of cross-validation, and (iii) provide a comprehensive source to inform study design decisions and recommendations for future basic research, anthelmintic efficacy trials, and resistance and clinical disease studies.

## Methods

### Literature search

An exhaustive literature search was conducted utilizing the University of Kentucky’s online InfoKat Discovery Library Catalog™ and Google Scholar. Additional publications were identified within references of relevant articles returned in searches or sourced from on-site archives of printed articles by corresponding authors affiliated with the Maxwell H. Gluck Equine Research Center.

Key search terms included strongyl*, small strongyl*, cyathostom*, trichonem*, gastrointestinal nematode, GIN*, parasit*, species, prevalence, abundance, survey, necropsy, equi*, horse, individual cyathostomin species names, and combinations thereof.

Inclusion criteria for literature returned in searches included peer-reviewed original article type published 1975 or later and available in English. Publications were necessarily available online, through interlibrary loan, within the M. H. Gluck Equine Research Center archive, or through direct communication with authors.

Studies were required to utilize domestic horses (*n* ≥ 4 individuals) and systematic cyathostomin adult collection methods categorized as (i) standard necropsies, (ii) critical tests, or (iii) diagnostic deworming. Standard necropsies were defined as *post-mortem* examinations, wherein adult specimens were recovered from digesta within the cecum, ventral colon, and dorsal colon *in toto* or from measured gut content aliquots, allowing estimation of total adult worm numbers. Critical tests were defined as studies in which horses were first anthelmintically treated, and adult specimens were collected from feces *in toto* or from measured aliquots for approximately five to seven days. After the post-treatment interval, horses were necropsied as above. By definition, diagnostic deworming required anthelmintic treatment of horses and specimen collection from post-treatment feces for one to three days post-treatment without subsequent *post-mortem* examination. Studies reporting incidental recovery of cyathostomins were excluded. A small number of controlled test anthelmintic efficacy studies in which standard necropsies were performed on treatment and control horses were returned in the literature search and were excluded.

Articles were required to cite cyathostomin adult morphologic identification keys with currently accepted species taxonomic designations and species assignment criteria (i.e. [1, 23–25]). The Lichtenfels et al. keys [1, 23] were generated and accepted by consensus of the equine helminthology research community following several World Association for the Advancement of Veterinary Parasitology (WAAVP) workshops and validated by the International Commission on Zoological Nomenclature (ICZN) [1]. The Tolliver key [24], amended by Kuzmina et al. [25], agrees with the Lichtenfels et al. keys, using a practical approach to reach the same identifications. Other keys yield outdated and incorrect species assignments, and studies using these were excluded. Relevant species omissions from the Tolliver key prior to amendment and implications thereof within this meta-analysis are discussed.

**Fig. 1:**
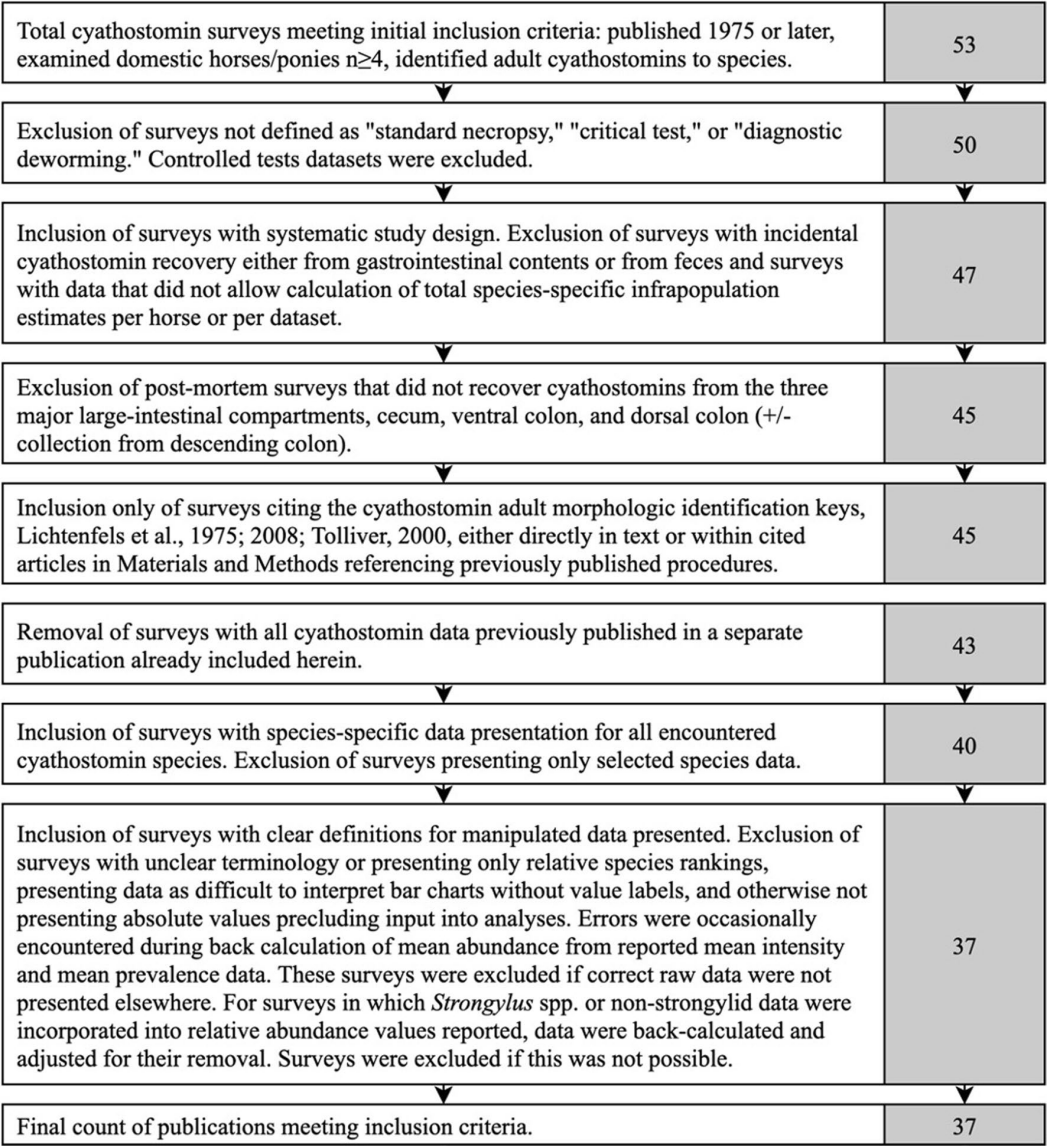
Flow diagram summarizing publications meeting stepwise inclusion/exclusion criteria

Ecological terminology recommended by Bush et al. [26], as pertains to parasitology, is employed herein. We carefully confirmed definitions of “abundance,” “relative abundance,” “intensity,” “count” (either of hosts or parasites), and “prevalence” within each publication. Publications wherein these terms were undefined, and raw data were not presented for clarification, were excluded. Studies were required to report data as prevalence and/or relative abundance for individual cyathostomin species recovered or raw data allowing calculation thereof. Mean prevalence and relative abundance were calculated per species across each dataset, when data permitted, for those publications in which they were not explicitly reported. A flow diagram summarizing the publication screening process is provided in Fig. 1.

### Statistical analyses

Statistical analyses were performed using SAS version 9.4 (Cary, North Carolina, USA). At the metapopulation level, the ‘GLIMMIX’ procedure for generalized linear mixed models was used to examine the influence of ‘Species’ main effect and interaction terms as well as species by region (‘Species*Region’) and species by specimen collection method (‘Species*Method’) effects on individual species grand mean prevalence and grand mean relative abundance across all respective datasets. Where possible, the interaction term species by region by specimen collection method (‘Species*Region*Method’) was also analyzed. The following parameters were used: link function, ‘Identity’; response variables weighted by number of hosts examined (‘HostN’) within datasets, and variance matrix blocked by dataset identifier (‘DataSetID’). Relative abundance data were square root transformed prior to analyses and back-transformed for data presentation. Pairwise comparisons of least squares means estimates (LSMs) with Tukey-Kramer adjusted *P*-values, and confidence interval limits (CL) were obtained for statistically significant effects. Significance was considered at α = 0.05 for all analyses. Negative prevalence and relative abundance estimates and confidence interval limits were interpreted as equal to 0%. Calculation of confidence interval widths for comparisons of variability between effects were calculated from back-transformed confidence interval limits with negative values retained.

The ‘Mixed procedure’ for mixed linear models was used to analyze the influence of ‘Region’ and ‘Method’ on the number of species reported (‘SpeciesN’) by prevalence datasets. Response variables were weighted by number of hosts examined, ‘HostN’. Pairwise comparisons were obtained as above and significance considered at α = 0.05 for all analyses.

## Results

### Literature search return

**Fig. 2:**
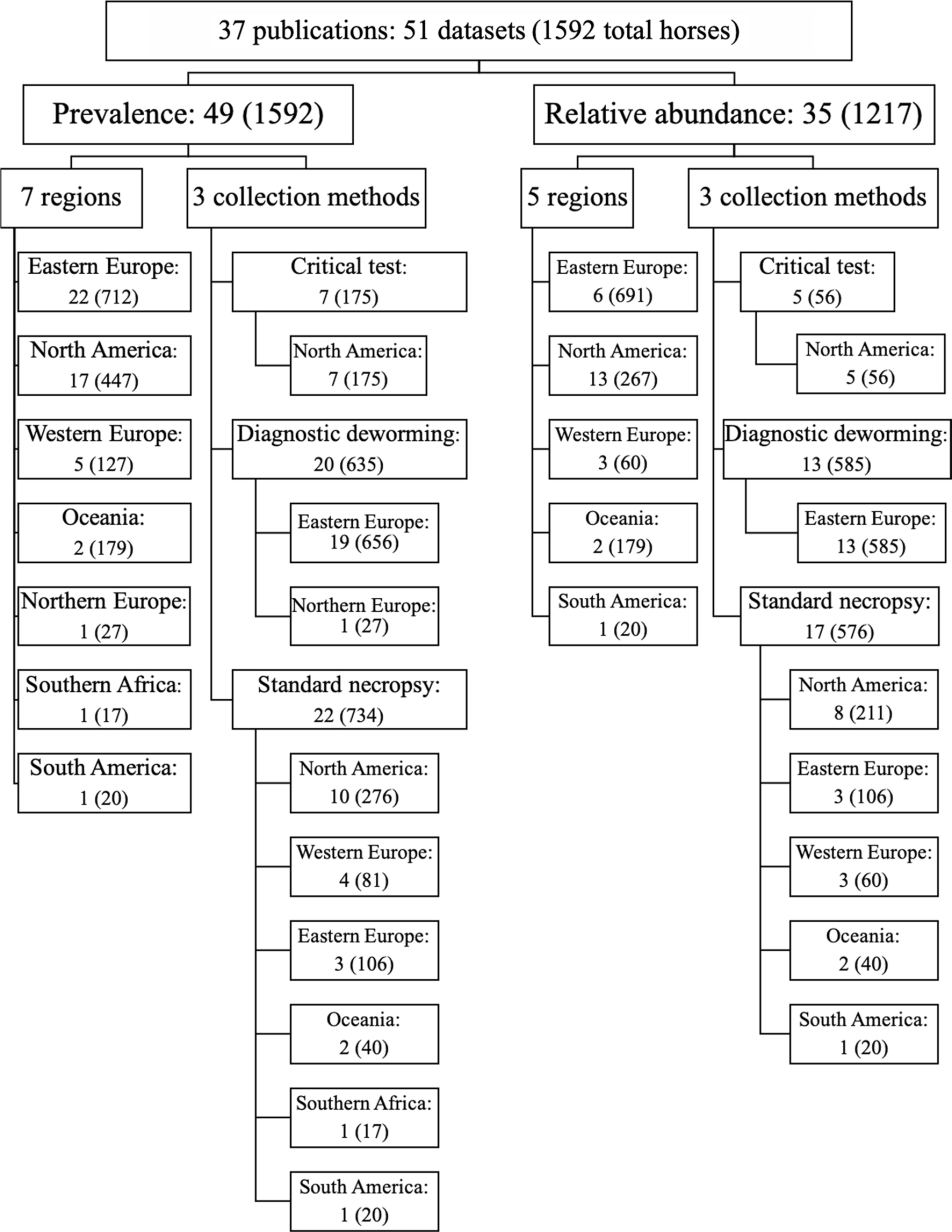
Flow diagram summarizing the number of data sets and horses included in the meta-analysis and a breakdown of prevalence and abundance data by region and collection method

Thirty-seven publications met the inclusion criteria, comprising 51 distinct datasets for which prevalence and/or relative abundance were reported or could be calculated for individual cyathostomin species. In one instance, data from two publications originating from the same group of horses were combined into one entry. Forty-nine datasets, utilizing 1592 equine hosts examined, yielded prevalence data, while 35 datasets, examining 1217 equine hosts, yielded relative abundance data (see Fig. 2 for an overview). Overall, 35 species of cyathostomins were reported. All included datasets were classified by study design type: standard necropsy (StndNcrp), critical test (CrtclT), or diagnostic deworming (DiagDwrm). Seven geographical regions were represented: North America (NAm), South America (SAm), southern Africa (SAfr), eastern Europe (EEur), western Europe (WEur), northern Europe (NEur) and Oceania (Ocea). All publication references and demographics are provided in Additional file 1: Table S1. Numbers of datasets and examined horses per region and collection method are summarized in Fig. 2.

### Arithmetic grand mean prevalence and relative abundance

**Fig. 3:**
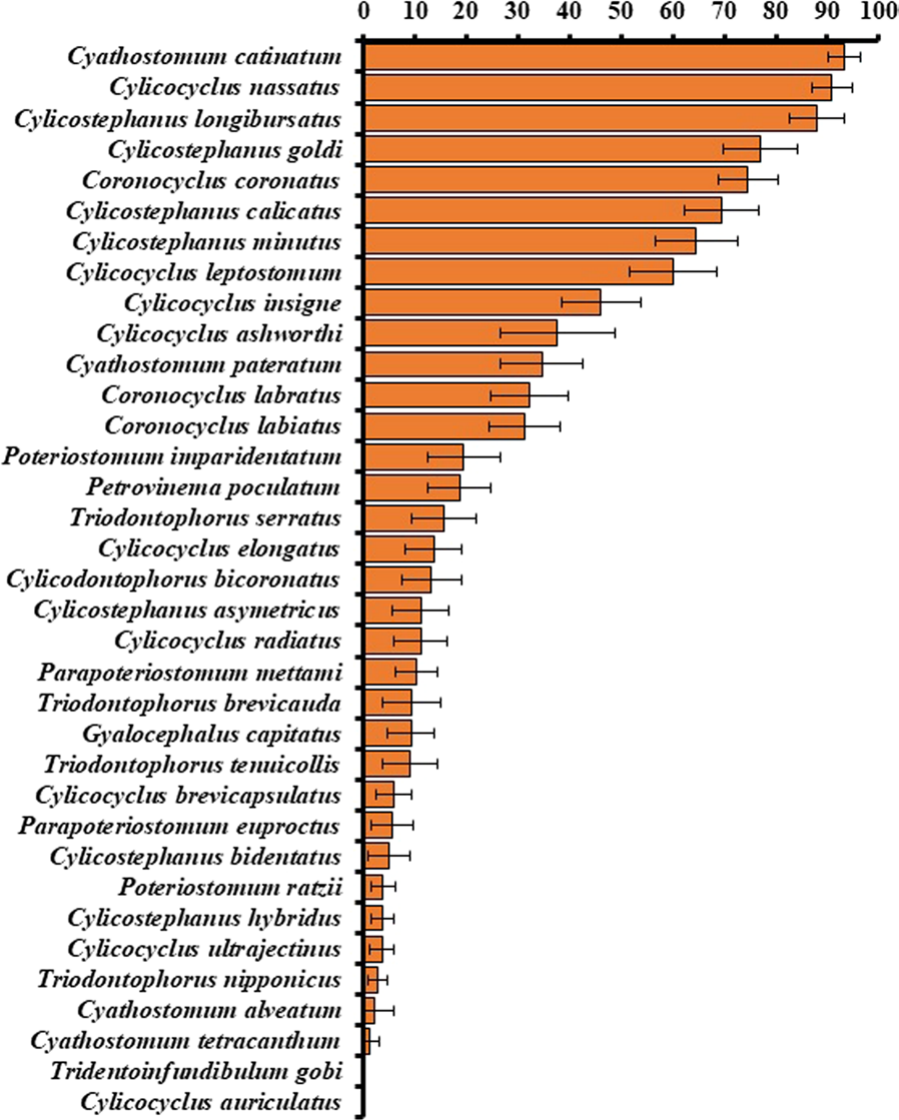
Grand arithmetic mean prevalence (%) ± 95% confidence interval for 35 cyathostomin species across 38 publications, 49 datasets, and 1592 equine hosts examined

**Fig. 4:**
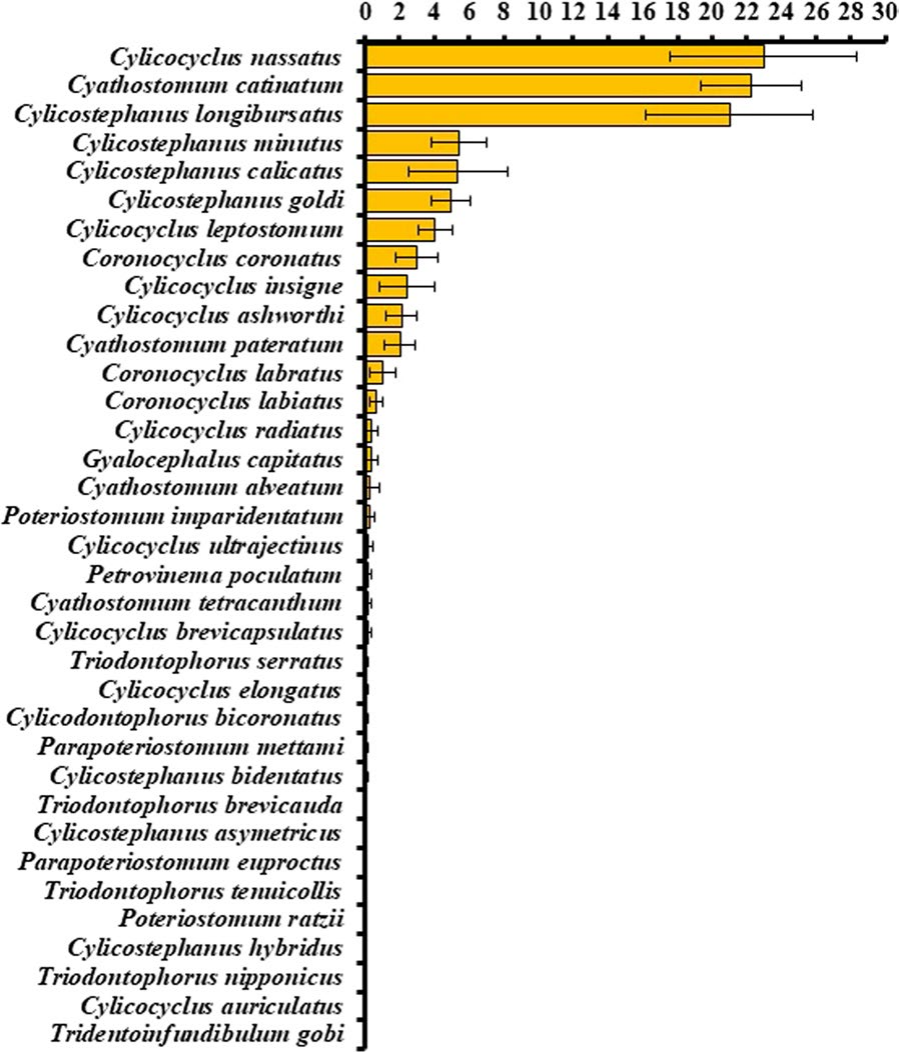
Grand arithmetic mean relative abundance (%) ± 95% confidence interval of 35 cyathostomin species across 29 publications, 35 datasets, and 1217 examined hosts

Grand mean prevalence and relative abundance with 95% confidence intervals for 35 cyathostomin species across 49 and 35 datasets, respectively, are presented in Figs. 3 and 4. The same three species, *Cyathostomum* (*Cya.*) *catinatum*, *Cylicocyclus* (*Cyc.*) *nassatus* and *Cylicostephanus* (*Cys.*) *longibursatus,* were the most prevalent (~88–93%) and with greatest relative abundance (21–23%) (Figs. 3 and 4). These three species together comprised ~ 66% of the total cyathostomin population across all datasets. The majority of species exhibited prevalence below 50% and relative abundance below 1%.

### Pairwise comparisons

*P-*values for main ‘Species,’ and interaction terms, species by region (‘Species*Region’) and species by specimen collection method (‘Species*Method’) effects on grand mean prevalence (%) least squares means estimates (LSMs) and grand mean relative abundance (%) LSMs are presented in Table 1. ‘Species’ and both interaction terms were significantly associated with both prevalence and relative abundance. Pairwise comparisons for all three effects follow. Some LSMs differed greatly from arithmetic means; thus, the analysis often organized species differently and grouped them more conservatively with adjusted data. This trend was evident in all pairwise comparisons, and noteworthy differences are detailed in the respective results subsections. All following main and two-way interaction pairwise comparisons are presented as Tukey–Kramer adjusted LSMs with confidence interval limits (CL), *P*-values, and conservative T groupings (T group). The T-group assignments designate whether estimates were statistically different from each other, in which case different letters are assigned. Effects of three-way interactions of ‘Species’ by ‘Region’ by specimen collection method (‘Species*Region*Method’) were limited to comparisons of two collection methods within or between NAm and EEur for the eight most prevalent and relatively abundant species. Three-way interaction data are presented as LSMs with 95% confidence intervals.

**Table 1:** Results from the meta-analysis of cyathostomin prevalence and relative abundance data

	Prevalence	Relative abundance
Species	**< 0.0001**	**< 0.0001**
Species*Region	**< 0.0001**	**< 0.0001**
Species*Method	**< 0.0001**	**< 0.0001**

*Notes*: *P-*values for ‘Species’ main effect and interaction terms, species by region (‘Species*Region’) and species by specimen collection method (‘Species*Method’) effects on grand mean prevalence and grand mean relative abundance least squares means estimates. Significant *P-*values in bold

#### Mean prevalence and relative abundance pairwise comparisons

Pairwise comparisons of species grand mean prevalence and grand mean relative abundance LSMs are presented in Tables 2 and 3, respectively. Some LSMs (*Cys. longibursatus* prevalence for example) differed greatly from respective arithmetic means. Species fell into four relatively distinct prevalence and relative abundance categories, characterized as ‘High’, ‘Medium’, ‘Low’ and ‘Very low.’ All categories were composed of the same species in both prevalence and relative abundance data, although respective species rankings within categories varied (Tables 2, 3). As with arithmetic means, *Cyc. nassatus*, *Cys. longibursatus* and *Cya. catinatum* were the most prevalent (~ 90–93%) and relatively abundant (~ 16–20%) species, and LSMs were not significantly different (Tables 2 and 3).

**Table 2:** Pairwise comparisons of prevalence (%) for 35 cyathostomin and selected strongylin species across 38 publications, 49 datasets, and 1592 equine hosts examined

	LSM	CL lower	CL upper	*P-*value	Group
Species categorized as ‘High’					
* Cylicocyclus nassatus*	93.4	90.5	96.3	**< 0.0001**	A
* Cylicostephanus longibursatus*	92.9	86.6	99.1	**< 0.0001**	A
* Cyathostomum catinatum*	90.6	87.1	94.1	**< 0.0001**	A
Species categorized as ‘Medium’					
* Cylicostephanus goldi*	81.8	77.0	86.7	**< 0.0001**	B
* Coronocyclus coronatus*	76.7	73.3	80.1	**< 0.0001**	B
* Cylicostephanus calicatus*	76.5	70.9	82.1	**< 0.0001**	B
* Cylicostephanus minutus*	71.4	65.8	77.1	**< 0.0001**	B, C
* Cylicocyclus leptostomum*	62.6	56.4	68.8	**< 0.0001**	C
Species categorized as ‘Low’					
* Cylicocyclus insigne*	36.0	30.3	41.7	**< 0.0001**	D
* Coronocyclus labratus*	31.0	24.9	37.2	**< 0.0001**	D, E
* Cyathostomum pateratum*	30.1	25.4	34.7	**< 0.0001**	D, E
* Coronocyclus labiatus*	28.7	24.4	33.1	**< 0.0001**	D, E, F
* Cylicocyclus ashworthi*	23.6	18.8	28.5	**< 0.0001**	E, F, G
Species categorized as ‘Very low’					
* Poteriostomum imparidentatum*	17.9	8.7	27.2	**0.0002**	E, F, G, H
* Cylicodontophorus bicoronatus*	16.3	12.9	19.6	**< 0.0001**	F, G, H
* Parapoteriostomum euproctus*	12.4	9.9	14.8	**< 0.0001**	F, G, H
* Petrovinema poculatum*	12.3	8.2	16.4	**< 0.0001**	F, G, H
* Triodontophorus tenuicollis*	12.0	4.1	19.9	**0.003**	F, G, H, I
* Cylicostephanus asymetricus*	11.6	5.8	17.3	**< 0.0001**	G, H, I
* Cylicocyclus brevicapsulatus*	11.1	8.2	14.1	**< 0.0001**	G, H, I
* Parapoteriostomum mettami*	11.0	6.3	15.7	**< 0.0001**	G, H, I
* Triodontophorus serratus*	7.9	3.0	12.8	**0.002**	H, I
* Cylicocyclus elongatus*	6.3	1.8	10.7	**0.006**	H, I
* Triodontophorus nipponicus*	6.1	5.5	6.7	**< 0.0001**	H, I
* Triodontophorus brevicauda*	5.7	1.2	10.3	**0.01**	H, I
* Cylicocyclus radiatus*	5.4	2.3	8.4	**0.0007**	H, I
* Poteriostomum ratzii*	5.3	4.2	6.4	**< 0.0001**	H, I
* Cylicocyclus ultrajectinus*	4.6	2.8	6.4	**< 0.0001**	H, I
* Gyalocephalus capitatus*	2.3	0.5	4.1	**0.01**	H, I
* Cylicostephanus hybridus*	1.0	0.1	1.9	**0.03**	H, I
* Cylicocyclus auriculatus*	0.1	0.05	0.1	**< 0.0001**	H, I
* Cyathostomum tetracanthum*	0	0	1.8	0.9	H, I
* Tridentoinfundibulum gobi*	0	0	0.05	0.1	H, I
* Cyathostomum alveatum*	0	0	0.5	0.4	I
* Cylicostephanus bidentatus*	0	0	1.2	0.3	I

*Notes*: Significance is considered at α = 0.05. Data are presented as Tukey-Kramer adjusted least squares means estimates (LSM), confidence interval limits (CL), *P-*values, and conservative T-statistic groupings (Group). Significant *P-*values in bold. Groups with same letters are not statistically different

**Table 3:** Pairwise comparisons of grand mean relative abundance (%) for 35 cyathostomin and selected strongylin species across 29 publications, 35 datasets, and 1217 equine hosts examined

	LSM	CL lower	CL upper	*P-*value	Group
Species categorized as ‘High’					
* Cylicocyclus nassatus*	20.31	16.53	24.47	**< 0.0001**	A
* Cylicostephanus longibursatus*	19.18	15.14	23.69	**< 0.0001**	A
* Cyathostomum catinatum*	16.39	14.15	18.80	**< 0.0001**	A
Species categorized as ‘Medium’					
* Cylicostephanus goldi*	5.98	5.07	6.97	**< 0.0001**	B
* Cylicostephanus minutus*	4.37	3.48	5.37	**< 0.0001**	B, C
* Cylicocyclus leptostomum*	4.04	2.56	5.87	**< 0.0001**	B, C
* Coronocyclus coronatus*	3.76	2.93	4.69	**< 0.0001**	B, C
* Cylicostephanus calicatus*	3.40	1.87	5.38	**< 0.0001**	B, C, D
Species categorized as ‘Low’					
* Cylicocyclus insigne*	2.08	0.95	3.63	**< 0.0001**	C, D
* Cyathostomum pateratum*	1.26	0.80	1.81	**< 0.0001**	D
* Coronocyclus labiatus*	1.18	0.71	1.76	**< 0.0001**	D
* Cylicocyclus ashworthi*	0.70	0.39	1.09	**< 0.0001**	D, E
* Coronocyclus labratus*	0.65	0.41	0.94	**< 0.0001**	D, E
Species categorized as ‘Very low’					
* Poteriostomum imparidentatum*	0.26	0.07	0.56	**< 0.0001**	D, E, F
* Cylicocyclus ultrajectinus*	0.15	0.03	0.34	**0.0002**	E, F, G
* Cylicocyclus elongatus*	0.12	0.05	0.24	**< 0.0001**	E, F, G
* Cylicodontophorus bicoronatus*	0.12	0.07	0.19	**< 0.0001**	E, F, G
* Cylicocyclus radiatus*	0.08	0.00	0.24	**0.01**	E, F, G
* Cylicocyclus brevicapsulatus*	0.05	0.00	0.18	**0.02**	E, F, G
* Poteriostomum ratzii*	0.04	0.01	0.09	**0.001**	E, F, G
* Parapoteriostomum mettami*	0.04	0.01	0.07	**< 0.0001**	E, F, G
* Parapoteriostomum euproctus*	0.03	0.01	0.05	**< 0.0001**	E, F, G
* Petrovinema poculatum*	0.02	0.00	0.09	**0.04**	E, F, G
* Triodontophorus serratus*	0.02	0.00	0.05	**0.02**	E, F, G
* Cyathostomum tetracanthum*	0.01	0.00	0.10	0.4	E, F, G
* Triodontophorus brevicauda*	0.01	0.00	0.02	**< 0.0001**	F, G
* Cylicostephanus bidentatus*	0.01	0.00	0.02	**0.001**	F, G
* Cylicostephanus hybridus*	0.00	0.00	0.01	**0.001**	F, G
* Cylicostephanus asymetricus*	0.005	0.00	0.01	**0.0004**	F, G
* Triodontophorus nipponicus*	0.004	0.00	0.01	**< 0.0001**	F, G
* Triodontophorus tenuicollis*	0.003	0.00	0.00	0.2	F, G
* Gyalocephalus capitatus*	0.0008	0.00	0.05	0.8	G
* Cylicocyclus auriculatus*	0.00004	0.00	0.00	**< 0.0001**	G
* Tridentoinfundibulum gobi*	0.00	0.00	0.00	**0.04**	G
* Cyathostomum alveatum*	0.00	0.00	0.00	0.4	G

*Notes*: Significance is considered at critical value, α = 0.05. Data are presented as Tukey-Kramer adjusted least squares means estimates (LSM), confidence interval limits (CL), *P*-values, and conservative T-statistic groupings (Group). Significant *P*-values in bold. Groups with same letters are not statistically different

#### Species by geographical region pairwise comparisons

**Fig. 5:**
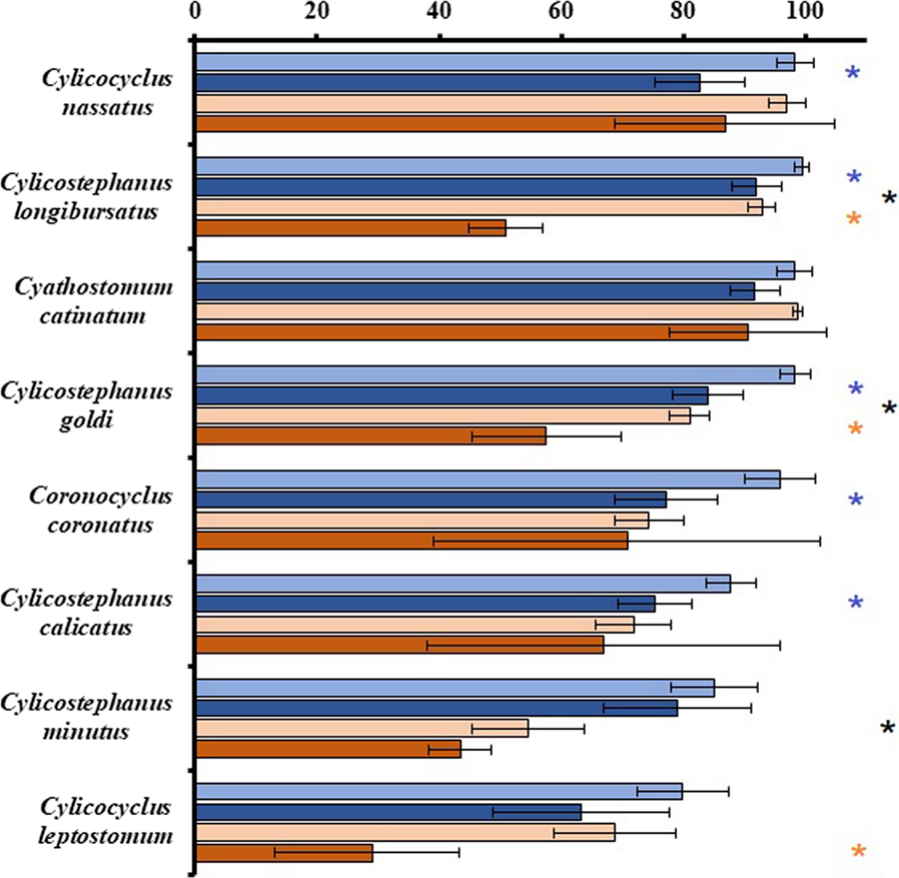
Species by region by collection method (‘Species*Region*Method’) pairwise comparisons of grand mean prevalence (%) for North America*Critical Test (light blue), North America*Standard Necropsy (dark blue), Eastern Europe*Diagnostic Deworming (light orange), and Eastern Europe*Standard Necropsy (dark orange). Data presented as least squares means estimates with 95% confidence intervals for the top eight most prevalent cyathostomin species. Asterisks denote significant differences (*P* < 0.05) in species prevalence: blue between North American methods, orange between eastern European methods, and black between North American and eastern European standard necropsies

**Fig. 6:**
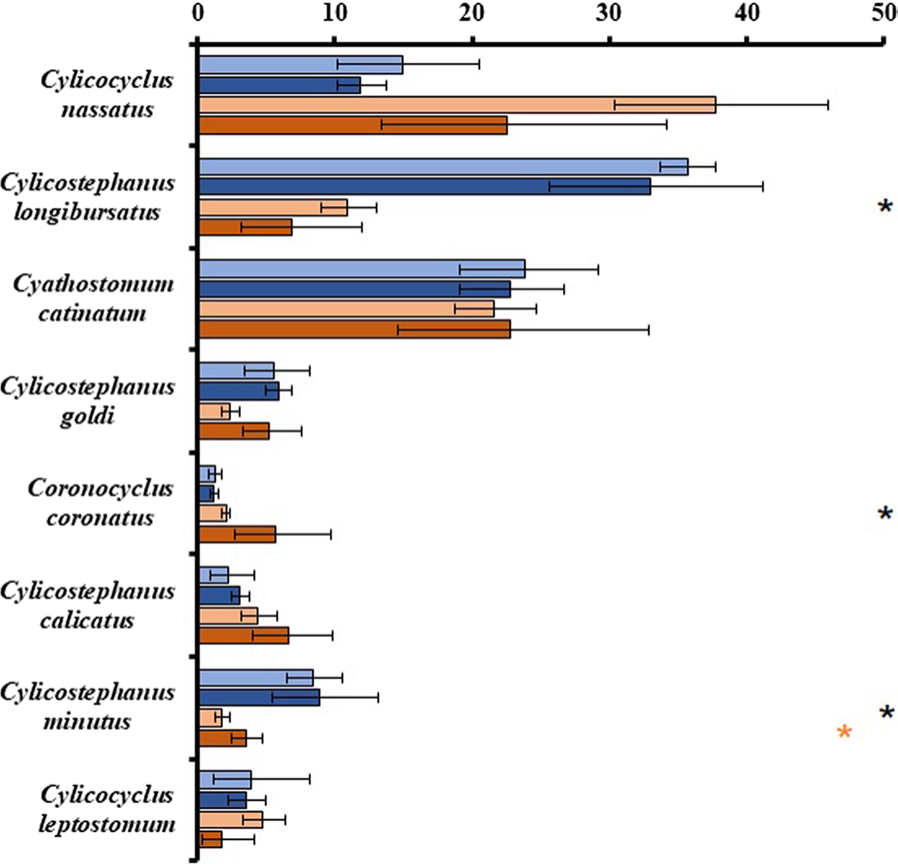
Species by region by collection method (‘Species*Region*Method’) pairwise comparisons of grand mean relative abundance (%) for North America*Critical Test (light blue), North America*Standard Necropsy (dark blue), Eastern Europe*Diagnostic Deworming (light orange), and Eastern Europe*Standard Necropsy (dark orange). Data presented as least squares means estimates with 95% confidence intervals for the top eight most relatively abundant cyathostomin species. Asterisks denote significant differences (*P* < 0.05) in species relative abundance: blue between North American methods, orange between eastern European methods, and black between North American and eastern European standard necropsies

Seven and five geographical regions were represented by prevalence and relative abundance datasets, respectively. Pairwise comparisons of species prevalence and relative abundance LSMs by ‘Region’ are presented in Additional file 2: Table S2 and Additional file 3: Table S3, respectively. Meaningful comparisons were between EEur, NAm, and WEur. On average, prevalence was most often highest in NAm. Prevalence and relative abundance LSMs for ‘High/Medium’ species in EEur, NAm, and WEur are presented in Figs. 5 and 6, respectively. *Cys. longibursatus* prevalence and relative abundance was significantly higher in NAm (~ 100%, 37%) than EEur (67%, 9%) (*P* < 0.0001). Prevalence of three additional ‘High/Medium’ species was significantly higher in NAm than EEur. Relative abundance for seven of the eight ‘High/Medium’ species was not significantly different between regions.

#### Species by specimen collection method pairwise comparisons

Species pairwise comparisons of grand mean prevalence and relative abundance LSMs by ‘Method’ are presented in Additional file 4: Tables S4 and Additional file 5: Table S5, respectively. For 17 species, prevalence was not significantly different between methods. For the six most prevalent species (prevalence > 76%, Table 2), StndNcrp yielded the lowest estimates and was significantly different from at least one other method in each instance. For all species, variability in prevalence based on confidence interval width was always lower in StndNcrp than respective variability within the other methods. In general, species relative abundance did not significantly differ between methods (*n* = 21 species). This was true for the three most relatively abundant species, *Cyc. nassatus*, *Cys. longibursatus* and *Cya. catinatum*. There were no instances for which a significant difference was found between all three methods. The confidence interval width for relative abundance LSMs within StndNcrp was generally less than or equal to the respective variability given by the other methods. Notably, confidence interval width for the top eight most relatively abundant species was between 1.5–4.9 times smaller in StndNcrp than in the other methods.

#### Species by regional specimen collection method pairwise comparisons

Breakdown of specimen collection method by region is presented in Figs. 5 and 6. Briefly, in both prevalence and relative abundance datasets, CrtclT were exclusive to NAm, DiagDwrm was performed primarily in EEur, and some StndNcrp occurred in both NAm and EEur. Due to localized use of CrtclT and DiagDwrm, three-way interaction effects, ‘Species*Region*Method’, were limited to pairwise comparisons of EEur*DiagDwrm | EEur*StndNcrp; NAm*CrtclT | NAm*StndNcrp; and EEur*StndNcrp | NAm*StndNcrp for the eight most prevalent and relatively abundant, ‘High’ and ‘Medium’ species.

Within NAm and EEur methods, prevalence was lower for all eight ‘High/Medium’ species in StndNcrp than in CrtclT or DiagDwrm. Differences were significant for five and three species in NAm and EEur, respectively. Within inter-region comparisons of StndNcrp, prevalence for all eight species, except *Cyc. nassatus*, was lower in EEur than in NAm, and differences were significant for three species. The most notable difference was the ~ 40 percentage point lower prevalence of *Cys. longibursatus* in EEur*StndNcrp (51%) than in EEur*DiagDwrm (93%, *P* < 0.0001) or NAm*StndNcrp (92%, *P* < 0.0001) (Fig. 5). Additionally, prevalence confidence interval widths were between 1.4 and 3.5 times larger in NAm StndNcrp than in CrtclT. Variability in EEur*StndNcrp prevalence was generally higher than in EEur *DiagDwrm with confidence interval widths ranging from 0.54 to ~ 17 times larger.

Relative abundance for three of the eight species was significantly different between NAm*StndNcrp and EEur*StndNcrp. Notably, the relative abundance of *Cys. longibursatus* was significantly lower in EEur*StndNcrp (7%) than in NAm*StndNcrp (33%) (*P* < 0.0001). Although non-significant, StndNcrp within both regions tended to yield lower estimates for the three ‘High’ species than the other method. This trend shifted in the five ‘Medium’ species, wherein StndNcrp yielded higher estimates than their regional counterpart. A relative abundance plot of ‘High’ ‘Medium’ and ‘Low/Very low’ species within the population total as given by regional specimen collection method is presented in Fig. 6. Standard necropsy resulted in larger contributions of ‘Medium’ and ‘Low/Very low’ species to the adult metacommunity than CrtclT and DiagDwrm, respectively.

#### Species richness pairwise comparisons

Species richness pairwise comparisons were only performed on prevalence survey data, as all but one relative abundance dataset was also represented by prevalence. Comparisons were additionally limited to regions represented by more than one prevalence dataset. Mean species richness by ‘Region’, ‘Method’ and ‘Region*Method’ are presented in Table 4. The average species richness ranged from 15 to 24 species. Notably, mean species richness in WEur (18) was significantly lower than in NAm (22) and EEur (23) (*P* < 0.0001), and EEur and NAm species richness was not significantly different (*P* = 0.4). Species richness within CrtclT (19) was significantly lower than in StndNcrp (21) (*P* < 0.0001).

**Table 4:** Mean species richness in prevalence surveys by ‛Regionʼ^a^ specimen collection method (‛Methodʼ), and region by collection method (‛Region*Methodʼ)

	LSM	Lower	Upper	Group
Region				
Oceania	24	23	25	A
Eastern Europe	23	23	24	A
North America	22	22	23	A
South America	22	–	–	–
Western Europe	18	17	19	B
Southern Africa	15	–	–	–
Northern Europe	15	–	–	–
Method				
Standard necropsy	21	20	21	A
Diagnostic deworming	20	19	21	A
Critical test	19	18	20	B
Region*Method interactions				
Eastern Europe*Standard necropsy	24	24	25	A
Eastern Europe*Diagnostic deworming	24	23	24	A
North America*Standard necropsy	23	23	24	A
North America*Critical test	21	21	22	B

^a^Pairwise comparisons between regions represented by more than one dataset; “–“ indicates no pairwise comparison

*Notes*: Pairwise comparisons are presented as Tukey-Kramer adjusted least squares means estimates (LSM), confidence interval limits (Lower and Upper), and conservative T-statistic groupings (Group). Groups with same letters are not statistically different

Within analysis of ‘Species*Region*Method,’ species richness did not differ between EEur methods, StndNcrp (24) and DiagDwrm (24) (*P* = 0.8), but was significantly lower in NAm*CrtclT (21) than in NAm*StndNcrp (23) (*P* < 0.0001). Species richness of StndNcrp was not significantly different across NAm and EEur (*P* = 0.5).

## Discussion

This is the first meta-analysis to consider equine cyathostomin species as comprising a greater metacommunity, describing community composition within the adult metapopulation infecting domestic horses around the world and the influence of region and adult specimen collection method on study outputs (i.e. species-specific prevalence and relative abundance and species richness) (Additional file 6: Dataset S1).

In this analysis, cyathostomin species were grouped into relatively distinct and consistently composed ‘High’ ‘Medium’ and ‘Low/Very low’ prevalence and relative abundance categories. Definitively, *Cylicostephanus* (*Cys.*) *longibursatus*, *Cylicocyclus* (*Cyc.*) *nassatus* and *Cyathostomum* (*Cya.*) *catinatum* were the most prevalent and relatively abundant species within the adult cyathostomin metacommunity, approaching 100% prevalence and comprising more than half of the adult metapopulation. The five species within the ‘Medium’ category; *Cys. goldi*, *Coronocyclus coronatus*, *Cys. calicatus*, *Cys. minutus* and *Cyc. leptostomum*, with prevalence > 50% should also be considered as common members of the metacommunity. Together, the top eight species comprised more than 75% of the total adult metapopulation. This validated the widely held assumption that natural mixed-infections consistently include 5–10 key species [6–8].

Localized use of critical tests in NAm and diagnostic deworming in EEur significantly limited our analyses, and this was further complicated by the fact that these studies were primarily performed by a single group in the USA (critical tests) and a single group in the Ukraine (diagnostic deworming). Nonetheless, these constrained comparisons yielded several interesting patterns. North American critical tests and EEur diagnostic deworming both produced higher respective prevalence estimates and lower variability thereof for the ‘High’ and ‘Medium’ species than standard necropsies conducted within the same respective regions, and differences were significant for several species (Fig. 3). The relative abundance data, although generally non-significant, suggested potential bias of NAm critical tests and EEur diagnostic deworming towards recovery of ‘High’ species, while respective regional standard necropsies gave more weight to ‘Medium’ and ‘Low/Very low’ species (Figs. 4 and 6). North American critical tests also yielded lower species richness estimates than respective standard necropsies (Table 4). These patterns were attributed to several major sources of both horse and cyathostomin sampling biases. Comparisons constrained to NAm and EEur standard necropsies provided limited evidence of regional differences; however, these comparisons were possibly confounded by further sampling biases between these standard necropsies.

We postulate that constraint of horse enrollment to female horses one year of age or older with detectable, patent cyathostomin infections resulted in consistent prevalence overestimation by NAm critical tests and EEur diagnostic deworming in relation to respective regional standard necropsies. By necessity, horses enrolled in NAm anthelmintic efficacy critical tests and in EEur diagnostic deworming studies generally had no or limited anthelmintic exposure immediately prior to enrollment and were prescreened for patent infections based on positive fecal egg counts [27–34], often meeting a predetermined threshold of 200 eggs per gram of feces (EPG) [35, 36]. In fact, all EEur diagnostic deworming horses from the 18 (of 19 total) datasets, for which pretreatment egg counts were reported, regardless of enrollment criteria, exhibited pretreatment fecal egg counts > 200 EPG [8, 31–33, 35–37]. Thus, cyathostomin prevalence in these horses was 100%, and, although there is no linear correlation of strongyle-type EPG with adult worm burden [38], enrolled horses had patent infections less likely to be negligible than horses with egg counts that were negative or below the threshold.

The included standard necropsies generally made fewer constraints on host enrollment, with some utilizing euthanized cases at veterinary hospitals or carcasses from slaughterhouses with varying ages and treatment histories [3, 6]. For the NAm standard necropsies, in particular, most horses were used from university research herds with little to no anthelmintic exposure [39–47], and a large number included foals well under six months of age with young, developing infections of low intensity and species richness [40–44]. Likewise, EEur standard necropsies ranged from opportunistic abattoir collection [3] to use of horses experimentally infected with naturally mixed cyathostomin larvae [48]. Thus, both NAm and EEur standard necropsies allowed inclusion of horses across the spectrum of infection intensity, decreasing mean species-specific prevalence and predisposing to high variability in comparison to respective regional critical tests and diagnostic deworming.

Additionally, the majority of NAm critical tests and EEur diagnostic deworming studies were performed at the component level; within each dataset individual horses were sourced from the same herd. In six of the seven NAm critical tests, horses were derived from two closed herds [30] with cyathostomin populations heavily selected for anthelmintic resistance to one or more anthelmintic classes [30, 49]. Likewise, diagnostic deworming utilized multiple, infected horses from the same herd within respective datasets [8, 25, 31–34, 37, 50]. This homogeneity of enrolled horses likely further limited variability in NAm critical tests and EEur diagnostic deworming studies. However, due to host sampling biases, we could not parse adult collection method effects and rule out the influence of host anthelmintic exposure levels on species-specific prevalence estimates.

North American critical tests and EEur diagnostic deworming studies estimated higher relative abundance of ‘High’ species and subsequently smaller contributions of ‘Medium’ and ‘Low/Very low’ species to the adult metapopulation than respective regional standard necropsies (Figs. 4 and 6). Due to the interdependence of prevalence and relative abundance terms, we expected a species-specific prevalence overestimation by NAm critical tests and EEur diagnostic deworming studies to be mirrored within relative abundance data. That this was not the case suggested NAm critical tests and EEur diagnostic deworming were biased towards recovery of abundant species and failed to recover ‘Medium’ and ‘Low/Very low’ species.

Similar species-specific relative abundance patterns in NAm critical tests and EEur diagnostic deworming studies implicated a bias associated with cyathostomin collection from post-treatment feces. Due to immense volumes of voided feces that expelled cyathostomins were distributed within, the probability that specimens were missed in the small daily fecal aliquots examined was high [20]. Incorporation of fecal examination within critical tests for estimating anthelmintic efficacy results in a conservative underestimation of initial efficacy [20]. However, using critical test data as estimates of species-specific prevalence and relative abundance within sampled component communities potentially lead to an inappropriate bias toward more abundant species, and uncommon species were more likely to be missed during recovery from feces [7, 20, 25]. Species infrapopulation totals were diluted further when only identifying a small percentage or predetermined number of the total specimens recovered [7] and again when adding these to the total number of specimens recovered at necropsy within critical tests to infrapopulation totals per horse. As diagnostic deworming relied solely on collection of anthelmintically susceptible specimens, we expected to see eastern European diagnostic deworming to obviously bias towards higher relative abundance of the most susceptible species, which should theoretically be the more uncommon species in comparison to EEur standard necropsies. We postulate that because we saw the converse; that the fecal collection bias toward common species outweighed and masked the possible bias exerted by the anthelmintic treatment used. Ultimately, the most abundant species exhibited sufficient anthelmintic susceptibility to be found in large numbers in post-treatment feces in EEur diagnostic deworming studies. As our analyses could not account for anthelmintic exposure and resistance levels, our interpretations are guarded. While fecal collection biased contribution of relative abundance categories to the adult metapopulation, the degree to which NAm critical tests produced these biases appeared less than that of EEur diagnostic deworming. This suggested that the incorporation of necropsy collection in critical tests dampened the real effects of fecal collection. Although differences in species category contributions to the total adult metapopulation seemed small, a shift of eight and 19 percentage points towards ‘Medium’ and ‘Low/Very low’ species totally in NAm and EEur standard necropsies, respectively, may be biologically significant. As 24 of the 35 cyathostomin species detected exhibited average relative abundance below 1%, these shifts could mean that relative abundance for more than half of the cyathostomin community was underestimated by NAm critical tests and EEur diagnostic deworming studies if values derived from respective regional standard necropsies are more accurate. Thus, specimen collection bias is a major caveat to inter-study comparisons, especially in surveilling effects of anthelmintic resistance.

Species richness values were also likely impacted by host and specimen sampling bias as well as major discrepancies in cyathostomin species identification keys employed. NAm critical tests yielded significantly lower species richness than NAm standard necropsies (*P* < 0.0001). The primary reason for this was almost certainly the use of an identification key in which three species were omitted. In the monograph, “A Practical method of identification of the North American cyathostomes (small strongyles) in equids in Kentucky, USA” *Cys. bidentatus* and *Cys. hybridus* were not recognized as cyathostomins infecting NAm horses [24]. This was amended in a later publication [25], which also acknowledged the misidentification of *Cys. bidentatus* as *Cys. asymetricus* in the Tolliver (2000) monograph [24]. Thus, *Cys. asymetricus*, *Cys. bidentatus* and *Cys. hybridus* were omitted from most NAm datasets utilizing the original monograph. Specimens labeled as *Cys. asymetricus* were not identified in any NAm standard necropsies and did not contribute to discrepancies in species richness between the two methods. However, one additional species, *Cyc. ashworthi*, a cryptic species of *Cyc. nassatus*, was not recognized as valid nor correctly identified by most cyathostomin taxonomists, including Tolliver [24], until well after publication of additional differential morphological characteristics [51] and molecular evidence validating these as distinct species [52]. *Cyc. ashworthi* has since been commonly found and often at high intensities in several countries including NAm [45, 46]. Thus, *Cyc. ashworthi* data are generally unreliable in earlier datasets, and prevalence and relative abundance of *Cyc. nassatus* may be overestimated. If *Cys. bidentatus*/*Cys. asymetricus*, *Cys. hybridus* and *Cyc. ashworthi* had been acknowledged, species richness in critical tests would have been ~ 24 and undoubtedly not different from NAm standard necropsies. This correction was justified as all of these species were later encountered either in individuals from the same herds used in the critical test datasets, in satellites of these original herds, or in other herds also maintained by the University of Kentucky Parasitology Group, where all critical tests occurred [53].

The similar corrected species richness between NAm critical tests and standard necropsies was surprising for several reasons. The first of these was the significant host and potential cyathostomin community homogeneity in critical tests. We investigated this further and observed that corrected species richness for critical tests performed on horses from the two herds heavily treated to select for resistant cyathostomin populations averaged to 23 [28–30, 54–56], while the corrected richness in the remaining critical tests was 28 [27]. Without overinterpreting these observations, this suggested that species richness in most North American critical tests was limited by high levels of selection pressure *via* anthelmintic treatment on these closed component populations as postulated by critical test study authors [57]. Species richness in NAm critical tests may also have been limited by sample size, as critical tests included only 56 horses, while NAm standard necropsies included 211. The number of species encountered increased with the number of specimens examined per horse [7] and the number of horses examined [8]. Similarly, authors of some eastern European diagnostic deworming studies observed reduced species richness between component communities and attributed this to historical anthelmintic exposure and anthelmintic resistance [31, 33–35, 37]. In our analyses, however, species richness in EEur diagnostic deworming studies was only slightly lower and not significantly different from that in EEur standard necropsy studies. As EEur standard necropsies included 106 horses and EEur diagnostic deworming studies included 537, standard necropsies may have somewhat underestimated the true regional species richness. In the absence of more robust EEur standard necropsy data, we could not dismiss the implication that most or all cyathostomin species in EEur exhibited some degree of susceptibility to the anthelmintics used in diagnostic deworming, and that diagnostic deworming performed with adequate host and cyathostomin sampling size was sufficient for accurate determination of species-specific presence/absence at the component level.

Despite potential influence of both horse and cyathostomin sampling biases, there was evidence of regional differences for at least two of the ‘High’ and ‘Medium’ species, *Cys. longibursatus* and *Cys. minutus*, substantiated by comparisons of NAm and EEur standard necropsies. Until specimen collection methods are cross-validated or localized methods are used across more regions to eliminate method biases, potential regional differences should still be considered in interstudy comparisons.

## Conclusions

Our analysis provides significant evidence that host and cyathostomin sampling biases critically affected published cyathostomin survey outputs (i.e. species-specific prevalence, relative abundance and species richness) and are major caveats to inter-study comparisons. Our data emphasize the importance of and need for standardization of study methods, data presentation, and accessibility for meaningful *post factum* analyses particularly in surveilling the development and spread of anthelmintic resistance and changes in cyathostomin species diversity. By collating published quantitative species-specific data, we provide a definitive source to inform these future recommendations for primary work on equine cyathostomins. Specifically, we intend our data to inform expansion of already published recommendations for minimum host and cyathostomin sample sizes for accurate community structure estimates with sufficient representation of uncommon and rare species. Our data also provide an opportunity to inform anthelmintic efficacy trial design recommendations, in which the eight ‘High’ and ‘Medium’ species present reliable targets for future species-specific efficacy determinations.

## Supplementary information

**Additional file 1: Table S1.** Publication and dataset demographics.

**Additional file 2: Table S2. **Pairwise comparisons of species prevalence (%) by region for 35 cyathostomin species within seven regions across 38 publications, 49 datasets, and 1592 hosts.

**Additional file 3: Table S3. **Pairwise comparisons of species relative abundance (%) by region for 35 Cyathostominae species within five regions across 29 publications, 35 datasets, and 1217 equine hosts examined.

**Additional file 4: Table S4.** Pairwise comparisons of species prevalence (%) by specimen collection method for 35 cyathostomin species for three methods across 49 datasets.

**Additional file 5: Table S5.** Pairwise comparisons of species relative abundance (%) by specimen collection method for 35 cyathostomin species for three methods across 35 datasets.

**Additional file 6: Dataset S1.** Complete data set from this study.

## Data Availability

All data analyzed during this study are included in this published article and its additional files.

## Abbreviations

NAm: North America
SAm: South America
EEur: Eastern Europe
WEur: Western Europe
NEur: Northern Europe
SAfr: Southern Africa
Ocea: Oceania
StndNcrp: Standard necropsy
CrtclT: Critical test
DiagDwrm: Diagnostic deworming
Cor.: *Coronocyclus*
Cya.: *Cyathostomum*
Cyc.: *Cylicocyclus*
Cyd.: *Cylicodontophorus*
Cys.: *Cylicostephanus*
Gya.: *Gyalocephalus*
Para.: *Parapoteriostomum*
Pet.: *Petrovinema*
Pot.: *Poteriostomum*
Trid.: *Tridentoinfundibulum*
Trio.: *Triodontophorus*
LSMs: Least square means
